# Words without meaning

**DOI:** 10.7554/eLife.54867

**Published:** 2020-02-21

**Authors:** Eve Marder

**Affiliations:** 1 Volen Center, Brandeis UniversityWalthamUnited States; 2 Biology Department, Brandeis UniversityWalthamUnited States

**Keywords:** Living Science, peer review, evaluation, language, mechanism, impact

## Abstract

Many of the words used by scientists when reviewing manuscripts, job candidates and grant applications – words such as incremental, novelty, mechanism, descriptive and impact – have lost their meaning.

When I was a child, we used to play a game of saying the same word over and over again until it lost all meaning. I don’t know why we found it so fascinating that incessant repetition resulted in sounds without semantics, but we did. Words can also lose their meaning if they are overused, or their meaning can change so that they become 'code' for something else in certain contexts.

Over the years I have grown to truly abhor some of the words that are overused and abused when we review manuscripts, job candidates, and grant applications. In particular, I now detest five words: incremental, novelty, mechanism, descriptive, and impact. These words are codes behind which we hide, and are frequently used in lieu of actual explanations of what people think about the subject at hand.

Where is it written that 'incremental' should be pejorative? Surely, all science is incremental at the limit, as everything we do benefits from past work. So, how large or small is a step forward that merits laud or scorn? This judgment depends totally on the perspective of the observer, and is probably one of most subjective judgments we make. When a reviewer uses the words 'novelty' or ‘novel’, these words are often code for 'I like this'. But, again 'novelty' is in the eye of the observer, and routinely manuscripts are termed incremental by one reviewer and novel by another.

Mechanism and descriptive are likewise often without meaning. 'Descriptive' is also commonly used as a pejorative, despite the fact that seeing a new phenomenon for the first time can be revelatory, and much important science is discovery through visualization and description. Indeed, it can be magically satisfying to see, for the first time under a microscope or with an electrode, something that no one had seen before, or perhaps even suspected was true. At times, one sees something in an image or a recording that changes in a moment how one thinks about a problem. Therefore, some of what can be called 'description' brings the observer, for the first time, intimately in touch with the biological system and its truths.

I frankly don’t know any longer what mechanism means. What is a biological mechanism? If you are working on processes of language comprehension, 'mechanism' might be nothing more than saying that a certain brain region contributes to the process; if you are studying seizure processes, it might mean that a particular cellular process is involved; and if you are investigating the release of synaptic vesicles, it might mean discovering the calcium-binding proteins. But, as we go down towards the cellular and molecular levels of analysis, the search for 'mechanism' is really a stand-in for the search for the underlying component processes. Where does this end? Each level of work that sheds light on mechanism according to one reader or reviewer might be dismissed as descriptive by another reader or reviewer. When a reviewer asks for mechanism in a paper or grant application, I suspect it often means no more than 'I want more because I personally am not satisfied with this level of explanation', but I still worry that it really means 'You have to do more just because you haven't done enough'.

However, my most detested word of the last decade is impact. According to the Google dictionary, the word impact has two meanings as a noun and two meanings as a verb. *noun* i) the action of one object coming forcibly into contact with another. ii) a marked effect or influence. *verb* i) come into forcible contact with another object. ii) have a strong effect on someone or something. For both the noun and the verb it is the second definition that has irreversibly altered our culture. My postdocs talk about wanting to publish a 'high impact paper', which is short-hand for publishing a paper in a journal with a high Journal Impact Factor. I want all of my people to aspire to do important work and to publish it well, but despite all my pleadings and blandishments, they have completely adopted 'impact' to denote their goals and remain convinced that publishing in a journal with a high Journal Impact Factor is synonymous with publishing the important papers that are necessary and sufficient for their futures.

**Figure 1:**
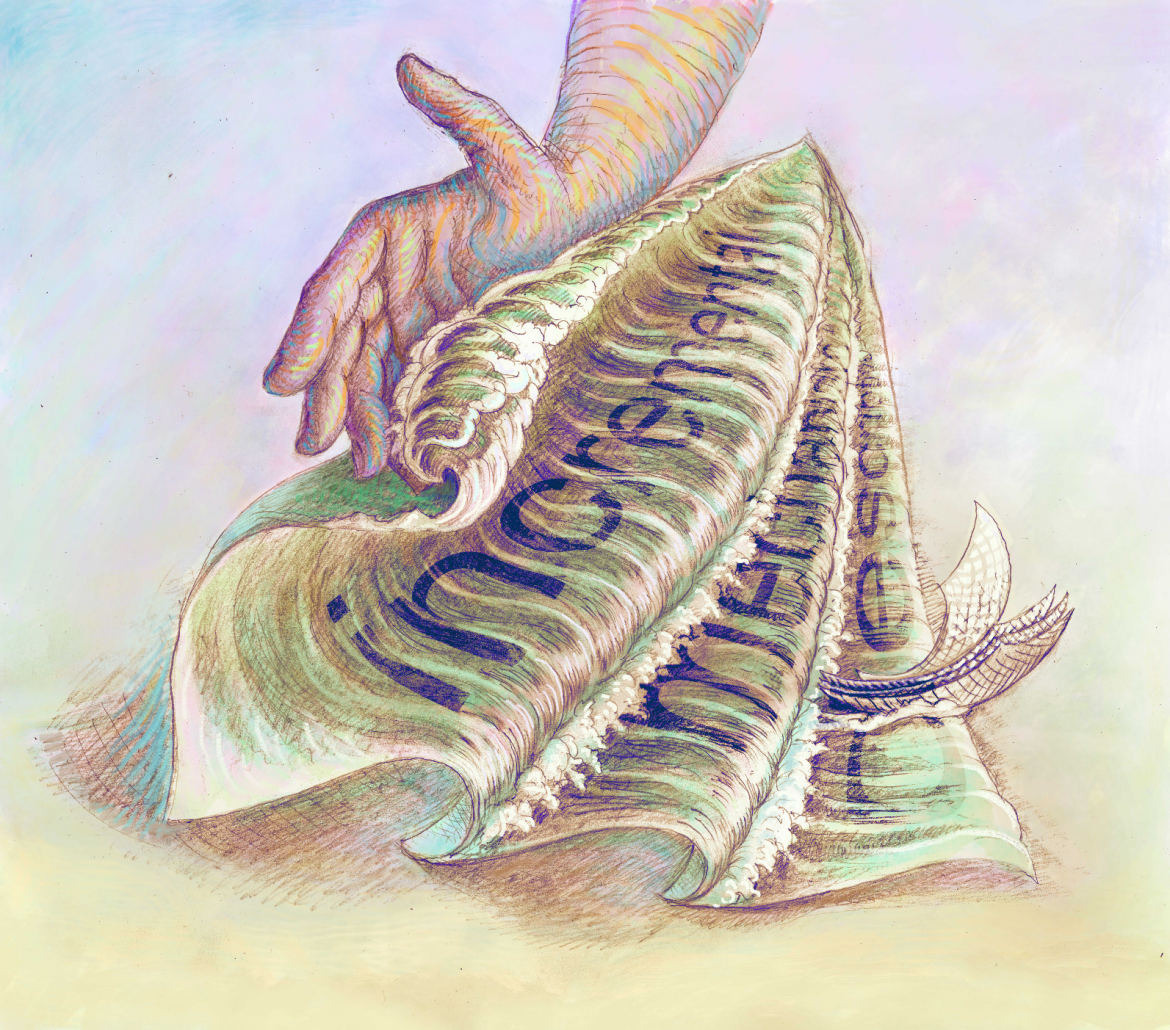
Many of the words used by scientists have hidden meanings.

Illustration: Ben Marder.

And it is hard to deny their reality: last month I wrote a letter in support of a promotion at an institution that required that the candidate provide, for each publication, the impact factor for the journal, its ranking in the field, as well as the number of citations of the actual paper. I was quite entertained to see how little correlation there was between the numbers of citations and the ranking of the journals!

But, my biggest complaint with 'impact' is when reviewers, grant panels, and selection committees believe that they can predict the future impact of the person and the work. Humans are notoriously poor at predicting the future, but somehow prognostications of future impact are made with gravitas, and are accorded undue validity. It continues to perplex me that scientists who are drawn to study the unknown seem so unwilling to live with the uncertainties that come from unpredictable future outcomes. Why do we find it so uncomfortable to admit that we are making guesses about the future significance of a given piece of work or the future success of a candidate? Of course, grant evaluation panels must make decisions about how likely they think it is that a proposed set of experiments will lead to new insight. But, we should all participate in these discussions and decisions with humility, and acknowledge to ourselves and each other that we are operating without the benefit of a crystal ball or a seer!

## Note

This essay is part of the Living Science collection.

